# The Impact of the Electric Field on Surface Condensation of Water Vapor: Insight from Molecular Dynamics Simulation

**DOI:** 10.3390/nano9010064

**Published:** 2019-01-04

**Authors:** Qin Wang, Hui Xie, Zhiming Hu, Chao Liu

**Affiliations:** Key Laboratory of Low-Grade Energy Utilization Technologies and Systems of Ministry of Education, School of Energy and Power Engineering, Chongqing University, Chongqing 400000, China; 20151001008@cqu.edu.cn (Q.W.); 20161013057@cqu.edu.cn (Z.H.); liuchao@cqu.edu.cn (C.L.)

**Keywords:** condensation, surface wettability, external electric field, molecular dynamic simulation

## Abstract

In this study, molecular dynamics simulations were carried out to study the coupling effect of electric field strength and surface wettability on the condensation process of water vapor. Our results show that an electric field can rotate water molecules upward and restrict condensation. Formed clusters are stretched to become columns above the threshold strength of the field, causing the condensation rate to drop quickly. The enhancement of surface attraction force boosts the rearrangement of water molecules adjacent to the surface and exaggerates the threshold value for shape transformation. In addition, the contact area between clusters and the surface increases with increasing amounts of surface attraction force, which raises the condensation efficiency. Thus, the condensation rate of water vapor on a surface under an electric field is determined by competition between intermolecular forces from the electric field and the surface.

## 1. Introduction

Vapor condensation is a widespread natural phenomenon which includes the formation of mist and rain [1], the production of droplets on a cooling surface [2], and so on. It is also an important process in industries such as thermal management [3], water desalination [4], and refrigeration [5]. Vapor condensation plays a fundamental role in high efficiency heat exchangers due to its ability to remove heat from systems like heat pipes [6] and industry reaction towers [7] by converting vapor into liquid. The vapor condensation in some systems can reduce the efficiency of the system. For example, the accumulation of condensed water on hot components during cooling can result in deteriorating performance and increasing cost.

Numerous studies have been performed on the behavior of vapor condensation to understand its mechanism under various conditions [8,9,10,11]. Condensation which occurs on a surface is more frequent than that found directly in the vapor phase because of the wide existence of solid substrate that can be seen as the nucleus. The properties of surfaces such as their structure and wettability can greatly affect condensation. Theories [12,13,14] and experiments [15,16,17,18] have demonstrated that condensation on a surface can be controlled by adjusting the wettability of the surface. Kripa et al. [15] have presented a method to achieve space control to enhance heat transfer in the condensation of water by using a combination of textured hydrophobic surfaces and hydrophilic tops. Cheng et al. [16] have investigated condensation in tubes and found that dropwise condensation can effectively improve heat fluxes and heat transfer coefficients. Zhang et al. [18] have used various coatings to observe condensation on hydrophobic surfaces. They have found that hydrophobic surfaces promote dropwise condensation effectively by limiting the growth of droplets. The effect of surface roughness on condensation and heat transfer has also been extensively studied [19,20,21]. Miljkovic et al. [20] have investigated the condensation of water on surfaces with different levels of roughness. They found that different morphologies of droplets formed on a surface can cause different amounts of heat flux. Mu et al. [22] observed condensation on magnesium surfaces and have revealed the relation between fractal dimensions and nucleation sites. Starostin et al. [23] have studied water condensation on superoleophobic and superhydrophobic surfaces and found that the depletion layer formed at the initial condensation stage on the substrate could affect the growth of condensation droplets.

Molecular dynamics simulations (MDS) can obtain detailed information at the molecular level, so MDS is popularly used to study the process of phase transition on surfaces at the nanoscale [24,25]. Xu et al. [26] have studied the nucleation of vapor on a *V*-shape surface by MDS and found the various angles of this *V*-type affected the state and nucleation rate of water droplets formed on the surface. Metya et al. [27] have studied the nucleation of water on nanoscale textured surfaces using MDS and have illustrated the dependence of nucleation rate on surface fraction. Toxvaerd [28] have studied vapor condensation on a planar surface with various attraction forces using MDS and have found that nucleation is promoted in the presence of the surface. Niu et al. [29] have used MDS to research the influence of wetting character on vapor condensation. In their study, they discovered that condensation on surfaces at the nanoscale is different from that in the bulk phase because the interfacial thermal resistance for hydrophilic surfaces is lower than that in the hydrophobic case.

Water exists widely in nature and industrial applications. The condensation of water has been found to be affected by temperature [30], supersaturation [31], electric field [32,33], and so on. Since water molecules are polar, an external electric field has a significant effect on the properties of water. Thus, electric fields have a wide range of industrial applications, such as electrospinning [34,35] and micro/nano-patterning [36]. Some simulations and experimental studies have been conducted to analyze the effect of an electric field on water characteristics. Butt et al. [37] have verified the Kelvin equation for the electric field experimentally and have proposed the relationship between electric field strength (*E*) and the pressure of condensing saturated gas. Zong et al. [38] have used MDS to study the viscosity of liquid water under different electric fields with various intensities and directions. They have found that both the electric field direction and strength could affect the viscosity of water. The wetting properties of water droplets on a surface can also be manipulated by the electric field due to the characteristics of water, including droplet spreading [39], droplet morphology transformation [40,41,42], and contact angle variation [43,44]. The potential application of an electric field on phase changes has also been studied. Aragones et al. [33] have studied phase changes of water under an electric field and have concluded that different phases of a phase transition occur under different intensities. Okuno et al. [45] have found that the application of an electric field in the direction perpendicular to the liquid-gas interface enhances evaporation, while application parallel to the gas-liquid interface impedes evaporation. Nandi et al. [46] have studied the crystallization of water within an electric field using MDS and have found that morphological shape transformation was important to realizing fewer defects and stacking faults.

The behavior of water molecules condensed on different wettability surfaces under an electric field and the effect of the combination of these two factors on condensation behavior have not been effectively investigated. Because of this, the present study uses MDS to investigate the condensation behavior of water molecules on different wetting surfaces in a uniform external electric field, including the condensation shape of water droplets on the surface, the orientation of water molecules, the density profile of water droplets, the condensation rate of water vapor, and the coupled effects of *E* and surface wettability on the condensation rate. Our results demonstrate a promising method to restraining the condensation of water on a surface.

## 2. Materials and Methods

**Figure 1:**
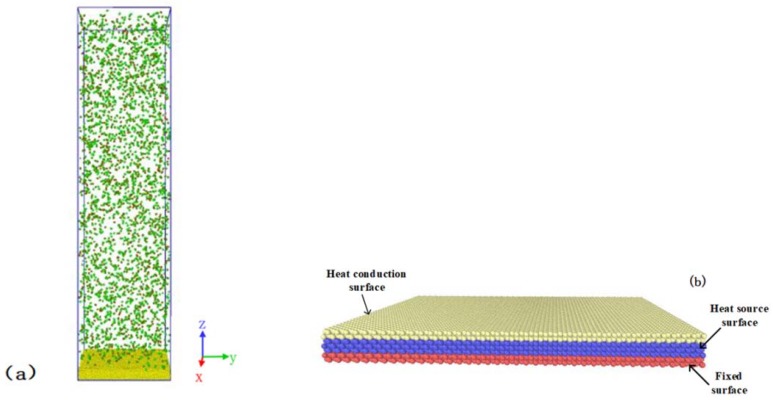
Configuration of the simulation systems. (**a**) Condensation of water molecules on the surface; (**b**) the structure of the surface for condensation.

All simulations presented in this work were performed with the LAMMPS package (2018) [47]. The condensation material used in these simulations was water. In order to study the effect of an electric field on condensation on a surface, we constructed a system comprising water molecules and a metal surface, as shown in Figure 1a. The surface included 12,000 Pt atoms. The embedded atom method (EAM) potential function [48] was applied to the surface atoms. The system contained 3200 water molecules and the TIP3P model [49] was employed for the water molecules. The TIP3P model specifies a three-site rigid-body molecule containing a Lennard-Jones interaction between an oxygen with three charges, with −0.834e for the O atom charge, +0.417e for the H atom, and the angle between the oxygen atom and the hydrogen atom being 104.52°. The TIP3P potential function, which includes Van der Waals interactions and Coulombic interactions, can be expressed as

(1) $$\omega =4\varepsilon_{ij}\left[{\left(\sigma /\gamma_{ij}\right)}^{12}-{\left(\sigma /\gamma_{ij}\right)}^{6}\right]+q_{i}q_{j}/4\pi \varepsilon_{o}\gamma_{ij}$$

where ε is the energy parameter, *σ* is the distance parameter, *i* and *j* represent different atoms, and *γ* is the distance between *i* and *j*. The second term indicates the interaction of Coulombic forces, *q_i_* and *q_j_* represent the charges of atoms *i* and *j*, respectively, and $\varepsilon_{o}$ represents the vacuum permittivity. The particle-particle particle-mesh (PPPM) method was adopted to compute long-range Coulombic forces. For oxygen atoms in the TIP3P model, *ε_O-O_* = 0.0044 eV and *σ_O-O_* = 3.188 Å. The interaction applied to the water molecules and the metal atoms was a 12-6 Lennard-Jones potential function in this simulation. The energy parameters *ε_O-Pt_* were modified in the range of 0.01 eV–0.02 eV to achieve hydrophobic and hydrophilic surfaces [50,51]. In order to save computational time, a cutoff radius of 12 Å was adopted for the Lennard-Jones potential and long-range Coulombic forces.

The solid surface was divided into three different surfaces, namely, a fixed surface, a heat source surface, and a heat conduction surface, as shown in Figure 1b. The fixed surface was located at the bottom of the entire surface and included atoms fixed at their equilibrium site to stabilize the system. The heat source surface faced the fixed surface, which was controlled by a Langevin thermostat to control the temperature of the surface. The heat conducting surface was in direct contact with the condensing substance (water). It transferred the thermal energy from the condensed water atoms to the heat source surface. The size of the simulation box was 12×12×48 nm^3^. Periodic boundary conditions were applied in the X and Y directions. The reflecting surface was applied in the Z direction so that the water molecules would always stay in the box.

In the equilibrium process, an NVT ensemble was used for the water molecules and the surface. To keep the water in the gaseous state, the temperature was maintained at 600 K using a Nose-Hoover thermostat. No electric field was applied throughout the process. After that, the NVE ensemble was used for the water molecules. The temperature of the heat source surface was maintained at 300 K to initiate a phase transition. The target electric field strength (*E*) was applied throughout the condensation process. The clusters were defined by the Stillinger criterion [52] which is that the distance between oxygen atoms in water molecules is less than 3.36 Å.

**Figure 2:**
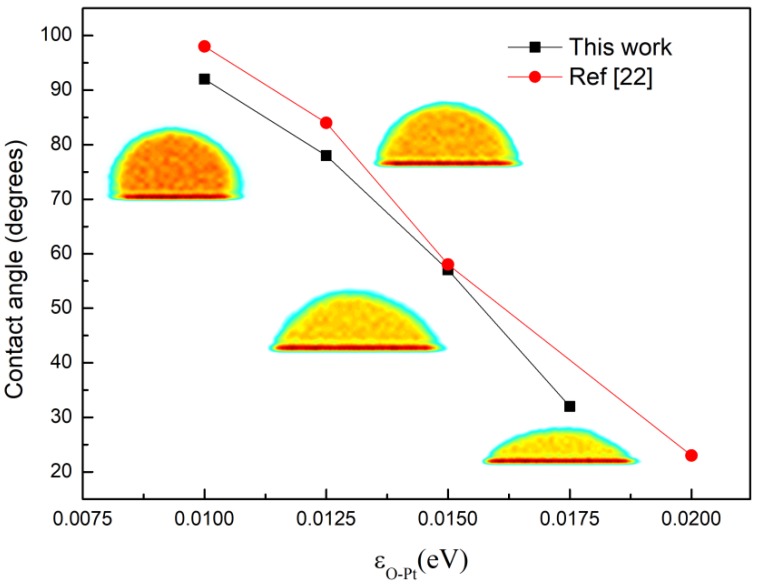
The contact angle of water droplets on surfaces with different wettability.

To acquire the wettability of the surface, we referred to the density contour of water droplets formed on the surface to confirm the contact angle of the droplets, which qualitatively characterized the wettability of the surface [53,54,55]. The parameters and molds were the same as in the condensation simulation. The variation in contact angle with wettability, which was controlled by *ε_O-Pt_*, is shown in Figure 2. The insets are the density contours of droplet corresponding to different energy parameters. It can be seen from the figure that the contact angle decreases gradually as *ε_O-Pt_* rises. This is consistent with other studies [29].

## 3. Results

### 3.1. The Effect of Electric Field Strength

**Figure 3:**
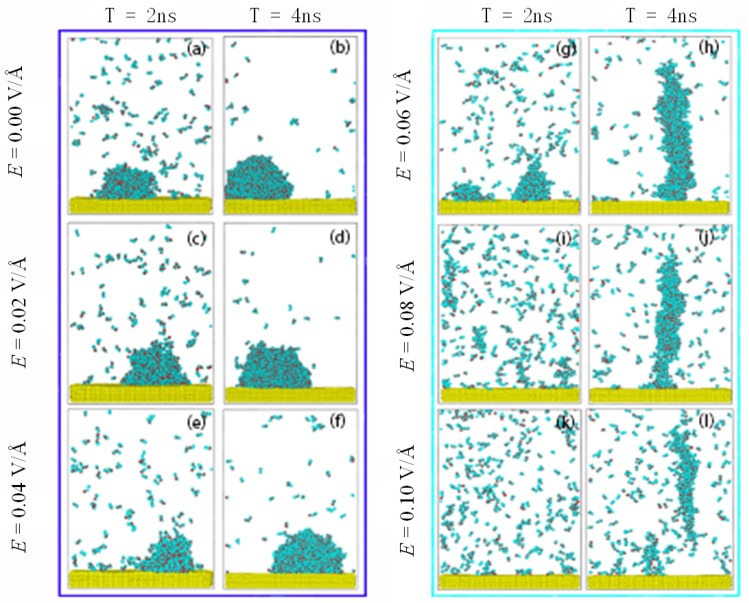
Snapshots of condensation of water molecules on the surface at 2 ns and 4 ns, under an electric field. (**a**) 2 ns, (**b**) 4 ns at *E* = 0.00 V/Å; (**c**) 2 ns, (**d**) 4 ns at *E* = 0.02 V/Å; (**e**) 2 ns, (**f**) 4 ns at *E* = 0.04 V/Å; (**g**) 2 ns, (**h**) 4 ns at *E* = 0.06 V/Å; (**i**) 2 ns, (**j**) 4 ns at *E* = 0.08 V/Å; (**k**) 2 ns, (**l**) 4 ns at *E* = 0.10 V/Å.

Figure 3 shows the condensation process of water molecules on the surface under different *E*. The condensation process of water on the surface is as follows. The high temperature water molecules near the surface will lose kinetic energy because of the temperature difference. The molecules will gradually deposit on the surface to form clusters. The clusters grow through gathering with other clusters and molecules. Droplets condensing on the surface form a spherical shape, as shown in Figure 3b, due to the interfacial tension.

**Figure 4:**
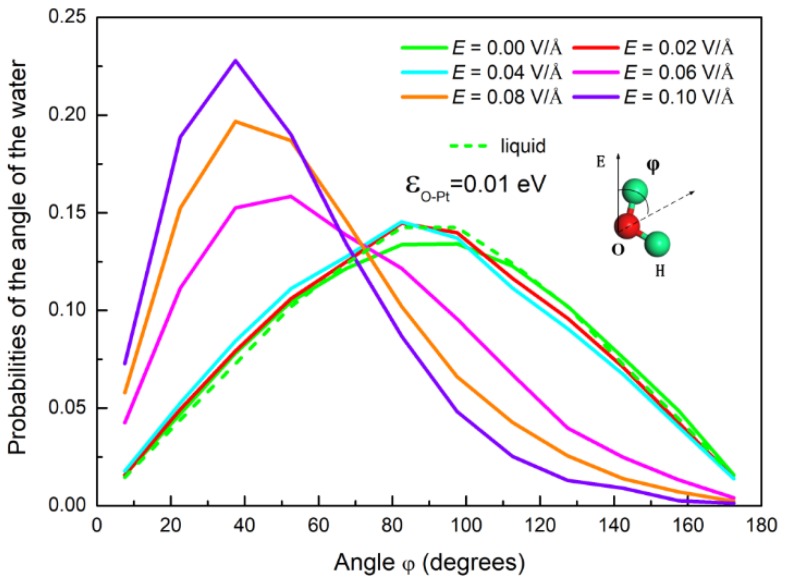
The probability distribution of the angle of the water molecules under different electric fields. The inset is a schematic for the angle *φ*.

When an electric field is applied to the system in the Z direction, the hydrogen atoms in the water molecules tend to point in the direction of electric field and the oxygen atoms in the opposite direction to the electric field. In this simulation, the molecules had a tendency to rotate upward under the electric field. It can be seen from Figure 3 that the clusters on the surface still held a spherical shape below 0.06 V/Å, indicating that the dominant forces were still the intermolecular forces among molecules rather than the electric force. When the *E* increased further, the electric force on the molecules became dominant, elongating the liquid droplets and causing the shape of the droplets to becomes a column, as shown in Figure 3h. Antonio et al. [42] have also observed the stretch of liquid water under an electric field in their experiments. This can also be confirmed by the molecular dipole distribution shown in Figure 4. When the electric field strength reached 0.1 V/Å, the condensed cluster separated from the surface. This has also been found in experiment [56].

As described above, condensation of water on a surface can be affected by the electric field due to the polarization that reorients the water’s molecular dipole moment. In order to characterize the polarization of water molecules, we calculated the angle *φ*, as shown in Figure 4. This was the angle between the dipole moment vector of the water molecules and the direction of the electric field. The probability distribution of the angle *φ* under different *E* is plotted in Figure 4. It can be discerned that, by comparison with the case without an electric field, the orientation of water dipoles in a weak field undergoes little change. The maximum value of the probability represents the dipole moment vector parallel to the surface. It is consistent with the results reported in the literature [41,57]. This indicates that the water molecules are still in a disordered state under a weak electric field. As *E* ≥ 0.06 V/Å, the peak of the angle distribution gradually changes to a smaller angle, which is also consistent with the literature [44,58]. This indicates that the electric force gradually makes more and more water molecules orientate their dipoles in accordance with the direction of the electric field. This results in a transformation from sphere to column, as shown in Figure 3.

**Figure 5:**
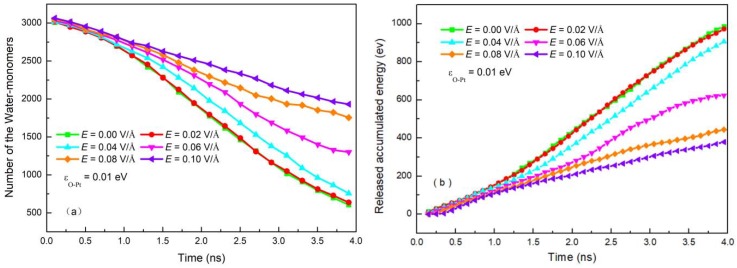
(**a**) The change of the number of monomers and (**b**) the released accumulated energy in the system with time under electric field.

The electric force on the water molecules can rotate the molecules, which not only causes a change in droplet morphology from sphere to column but also affects the condensation of water on the surface, as seen in the snapshots of condensation in Figure 3. To further understand the effect of an electric field on condensation on a solid surface, we calculated the number of monomers that did not bond with others retained in the system over time, which characterizes the condensation processes under different *E*. The results are shown in Figure 5a.

Condensation on a surface is determined by competition between the forces caused by the electric field and the attraction force from the surface. An electric field can rotate water molecules upwards with the torsion force, as described above. Therefore, this force can partly offset the attraction to the surface. The stronger the electric field, the bigger the torsion force, meaning the effect of attraction from the surface on condensation becomes less. In other words, the number of monomers increases as *E* increases, as shown in Figure 5a. When *E* ≥ 0.06 V/Å, the shape of clusters on the surface changes. This means that the torsion force coming from the electric field becomes the dominant one. Thus, the impediment of the electric field on condensation becomes more obvious.

The condensation process is an exothermic process and the energy of condensation matter will be released during the condensation process. A determined factor for condensation is the rate of heat exchange between condensation matter and a cold surface. Thus, the effect of *E* on condensation can be reacted from the energy of condensation matter. Figure 5b shows the evolution of the accumulation heat from water vapor during the condensation process. The slope of each curve is the heat flux. It is clear that the heat flux decreases with increasing *E*. When *E* ≥ 0.06 V/Å, the heat flux declines rapidly. This is because the stronger *E* makes the shape of the clusters change from a sphere to a column when the electric force starts to dominate. The results from Figure 5 indicate that a vertical electric field can restrain condensation, especially the intensity for the shape transformation.

**Figure 6:**
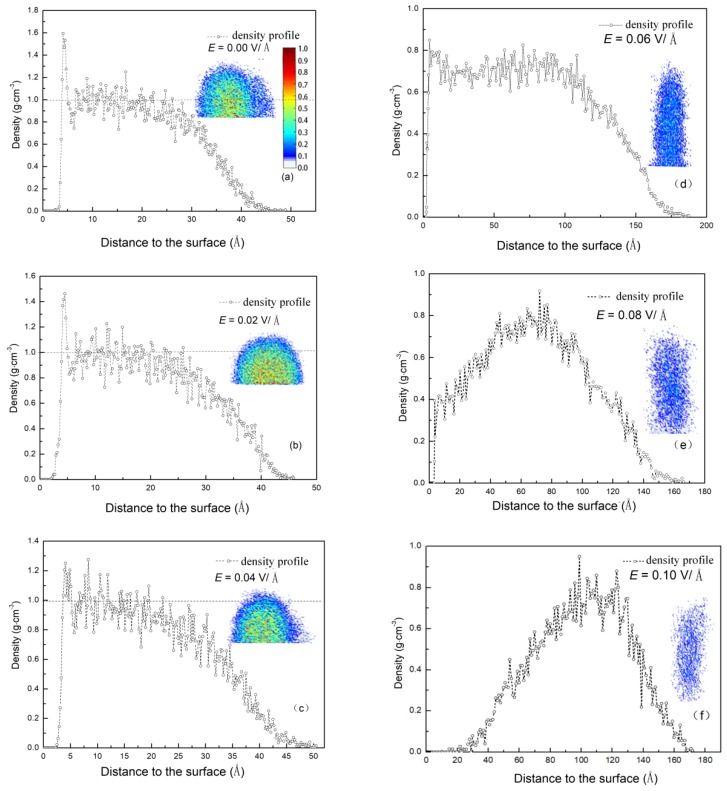
The density profiles of the largest cluster under different electric field strength along the centerline of the *Z* axis. (**a**) *E* = 0.00 V/Å; (**b**) *E* = 0.02 V/Å; (**c**) *E* = 0.04 V/Å; (**d**) *E* = 0.06 V/Å; (**e**) *E* = 0.08 V/Å; (**f**) *E* = 0.10 V/Å; The inset figures show the density contours, which is the middle cut of the largest cluster in the X-Y plane.

The driving force for condensation is the temperature difference between mutual contact materials. In addition, the torsional force, caused by the electric field and exerted on the molecules, can influence water molecules to come into contact with substances. The temperature difference exists not only between the surface and the water vapor but also among the water molecules during condensation. Therefore, the efficiency of energy exchange affected by the electric field can be analyzed using two factors. The first is the number of water molecules near the surface, which determines the efficiency between the water molecules and the surface. The second is the structure of the formed clusters, which determines the efficiency among water molecules. In order to understand the effect of an electric field on the condensation, the density distribution of the clusters along the centerline of the *Z* axis was calculated. The results are shown in Figure 6. The density contours of clusters condensed on the surface under different electric field strengths are shown in the insets. It can be seen from the figure that there is a lower density domain at the edge of the clusters. The lower density domain consists of thin layers of absorbed liquid molecules, which can be called precursor films. This phenomenon has also been found in experiment [23]. The dotted horizontal line in the figures corresponds to the bulk density. With increasing *E*, the clusters were stretched by the electric force and the density decreased gradually. When the electric force *E* < 0.06 V/Å, the water molecules remained tightly bound together. However, as the electric field force dominated, the water molecules were rearranged by this force and the bulk density region disappeared. With the force further increasing, the amount of surface attraction reduced, causing the clusters gradually formed in the environment. This resulted in the reduction of density adjacent to the surface. Thus, the electric field impedes water molecules from coming into contact with the surface, leading the process of condensation to become slow.

In summary, the torsional force, caused by the electric field, causes water molecules to rotate. This can result in two changes. Firstly, the force partly offsets the attraction force from the surface. Secondly, the force causes the area of clusters that are in direct contact with the surface to decrease and the structure of the clusters to become loose. Hence, the vertical electric field can restrain the condensation of vapor on the surface and the effect of restriction of the electric field becomes obvious as the shape of the clusters transforms from a sphere to a column.

### 3.2. The Coupled Effect of Wettability and Electric Field

The condensation of water molecules under an electric field is affected by intermolecular forces among water molecules, the attraction force of the surface, and the electric field force. In the above section we discussed the effect of *E* on condensation. Now, we investigate the coupled effect of attraction force from the surface and *E*.

**Figure 7:**
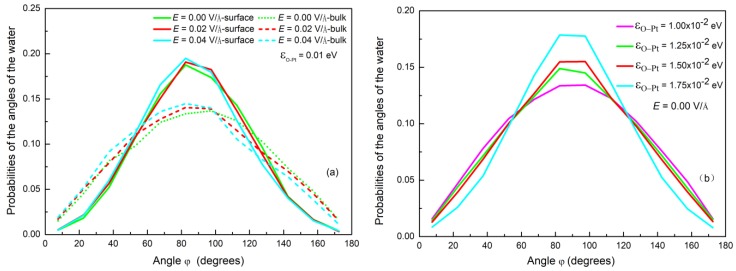
(**a**) The probability distribution of *φ* near the surface and in the bulk; (**b**) the probability of *φ* on different wettability surface with no electric field.

**Figure 8:**
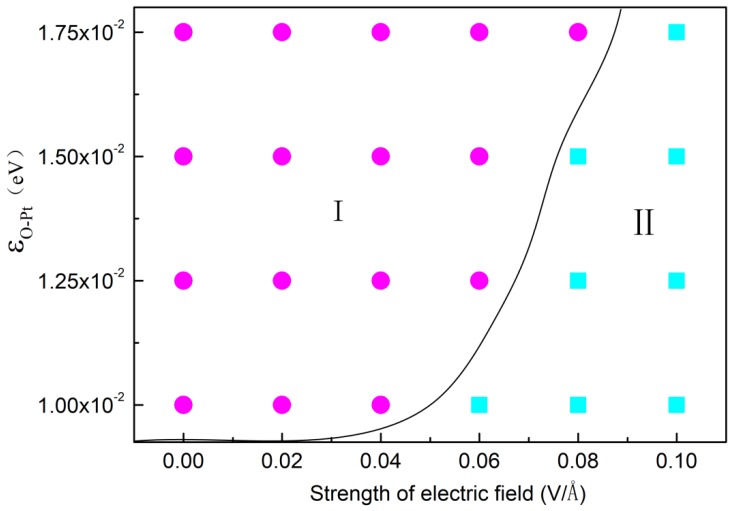
The configuration of cluster forming on different wettability surface under different electric field, where square represents circular cluster, circular represents columnar cluster.

The presence of the surface not only provides a cold source but it also influences the effect of electric forces on water molecules during condensation. Figure 7a shows the probability distribution of the angle *φ* of water molecules near the substrate and in the bulk, where the potential energy $\varepsilon_{O-\mathrm{Pt}}$ is 0.01 eV. It can be found that the distribution of angles in the bulk phase is lower than that near the surface. This indicates that the surface hinders the deflection of the water molecules near the surface and decreases the influence of the electric field on reorientation. Figure 7b shows the probability distribution of the angle *φ* for cases with different wettability and without an electric field. The distribution is symmetrical and the number of molecules with dipole moment vectors parallel to the surface increases as $\varepsilon_{O-\mathrm{Pt}}$ rises. It can be deduced from Figure 8 that the surface will restrain the rotation of water molecules under an electric field [59] and that this effect is enhanced as $\varepsilon_{O-\mathrm{Pt}}$ increases.

As analyzed above, water molecules in an electric field can be rotated by the electric force but the presence of a surface obstructs this and this trend is more evident as *ε_O-Pt_* increases. Figure 8 displays the morphology of droplets on different surfaces with different *ε_O-Pt_* under different *E*. The squares in the figure mean that the condensation cluster has transformed into a column and the circles mean it is still a spherical droplet. It may be deduced that condensation on the surface is influenced by competition between the electric field force and the attraction force of the surface. In other words, the electric field force, required for structural transformation, increases as *ε_O-Pt_* increases. When the value of *ε_O-Pt_* is 1.00 × 10^−2^ eV, the *E* needed to form column-shaped clusters is 0.06 V/Å. When *ε_O-Pt_* = 1.75 × 10^−2^ eV, the *E* needed rises to 0.10 V/Å.

**Figure 9:**
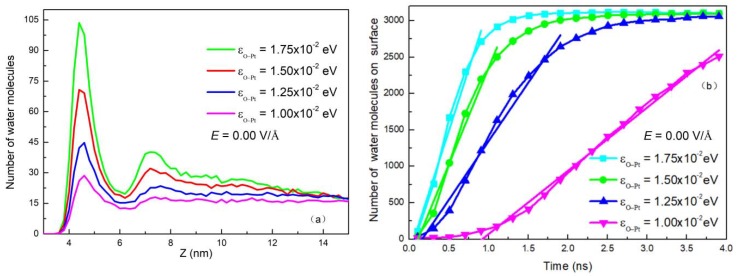
(**a**) Distribution of water molecules in a droplet along the *Z*-axis and (**b**) The number of water molecules deposited on the surfaces as a function of time with different wettability in the absence of electric field.

Change in surface wettability alters attractive force to determine the distribution of water molecules on the surface. Figure 9a shows the distribution of water molecules in clusters on the surface along the *Z* axis with no electric field. It can be seen from the figure that as *ε_O-Pt_* increases, there are more water molecules attracted to the surface. This increase in water molecules near the surface improves the efficiency of heat exchange. Figure 9b shows the relationship between the number of water molecules condensed on the surface and the time without electric field under different surface wettability. It is apparent that as *ε_O-Pt_* increases, the amount of water molecules condensed on the surface increases at the same time in the linear growth stage, where the slope is defined as *V_c_*.

**Figure 10:**
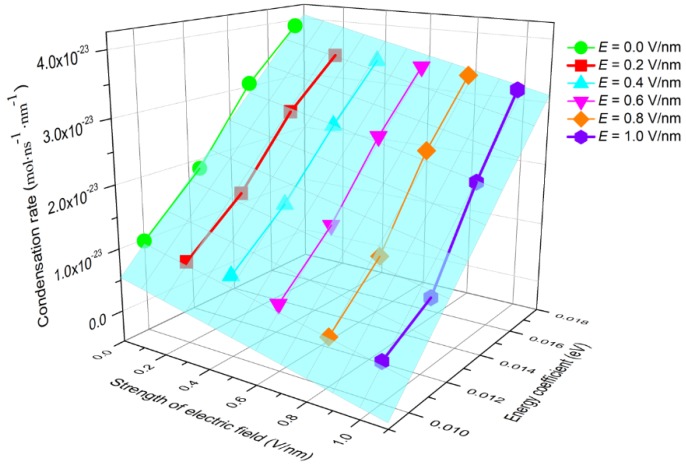
The condensation rate with different wettability in the electric field.

The condensation of water molecules is affected by the electric field force and surface wettability, as demonstrated in Figure 5 and Figure 9. In order to quantitatively characterize the coupled effect of these two factors on condensation, the condensation rate, *V_c_*, under different *E* and *ε_O-Pt_*, was calculated. The results are shown in Figure 10. The relationship of *V_c_* to *E* and *ε_O-Pt_* can be described well by the function

(2) $$V_{c}(\varepsilon_{O-Pt},\;E)=\alpha \times \varepsilon_{O-Pt}+\beta \times E+\gamma$$

which fits the data from this simulation and where $\alpha =3.91\times 10^{-21}\;\mathrm{mol}/\left(\mathrm{ns}\cdot \mathrm{eV}\cdot {\mathrm{nm}}^{2}\right)$, $\beta =-8.63\times 10^{-24}\mathrm{mol}/\left(\mathrm{ns}\cdot V\cdot {\mathrm{nm}}^{2}\right)$, and $\gamma =-2.98\times 10^{-23}\;\mathrm{mol}/\left(\mathrm{ns}\cdot {\mathrm{nm}}^{2}\right)$. The coefficient errors are ±5.83%, ±9.73%, and ±15.74%, respectively. It can be seen from the figure that as *E* increases and *ε_O-Pt_* decreases, *V_c_* reduces gradually, that is, the condensation rate is positively correlated with the wettability of the surface and is negatively correlated with *E*.

To further illustrate the coupled effect of electric field and *ε_O-Pt_* on *V_c_*, we calculated the ratio *f*, which is defined as

(3) $$f=\left(V_{non}-V_{ef}\right)/V_{non}$$

**Figure 11:**
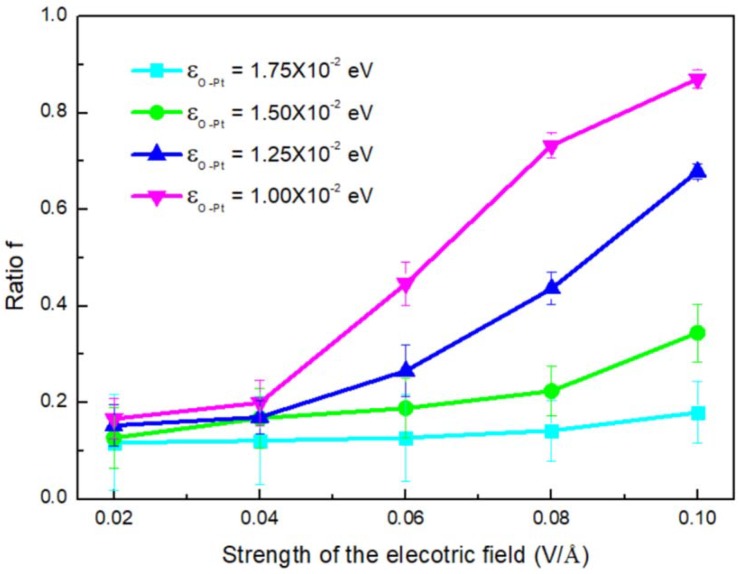
The ratio (*f*) varies with the strength of the electric field, where the deposition rate for the systems with and without electric field (*V_ef_*).

It was used to elucidate the difference between the condensation rates for systems with an electric field (*V_ef_*) and without an electric field (*V_non_*) under different conditions. The results are shown in Figure 11. As *E* increased, *f* became larger and larger; it also increased as *ε_O-Pt_* increased. By comparing Figure 11 with Figure 10, it can be found that when the shape of the clusters transforms to a column, the effect of the wettability of the surface on *f* is more obvious. Surface wettability tending to hydrophilicity can effectively increase the condensation rate of water molecules but the efficiency can be restrained by the electric field.

## 4. Conclusions

The condensation of water vapor on surfaces under an external static electric field has been studied here by using molecular dynamics simulations. We analyzed the coupling effect of electric field strength and surface wettability on condensation. The presence of electric field exerts a torsional force on molecules so that water molecules are rotated along the direction of the electric field. The rearrangement increases with increases in intensity and conclusively transforms the morphology of clusters from spherical to columnar. In addition, in accompaniment to the shape varying, the density of clusters near the surface decreases. This leads to the contact area between the condensation clusters and the solid substrate decreasing and the suppression of the energy exchange. Thus, the condensation rate becomes slower and slower as the electric field intensity increases.

When the surface wettability changes from hydrophobicity to hydrophilicity, it prevents molecule rotation near the surface and increases the contact area between the surface and clusters. It causes the intensity of the electric field, needed for the transition of the cluster shape, in the system with hydrophilic surfaces to be stronger. In addition, the condensation efficiency increases with the solid-liquid interaction intensity. The condensation rate within the electric field is determined by competition between the intermolecular forces from the electric field and the surface.
